# Heterologous Expression of Cyclodextrin Glycosyltransferase *my*20 in *Escherichia coli* and Its Application in 2-*O*-α-D-Glucopyranosyl-L-Ascorbic Acid Production

**DOI:** 10.3389/fmicb.2021.664339

**Published:** 2021-05-28

**Authors:** Kai Song, Jingjing Sun, Wei Wang, Jianhua Hao

**Affiliations:** ^1^College of Food Sciences and Technology, Shanghai Ocean University, Shanghai, China; ^2^Key Laboratory of Sustainable Development of Polar Fishery, Ministry of Agriculture and Rural Affairs, Yellow Sea Fisheries Research Institute, Chinese Academy of Fishery Sciences, Qingdao, China; ^3^Laboratory for Marine Drugs and Bioproducts, Qingdao National Laboratory for Marine Science and Technology, Qingdao, China; ^4^Jiangsu Collaborative Innovation Center for Exploitation and Utilization of Marine Biological Resource, Lianyungang, China

**Keywords:** 2-*O*-α-D-glucopyranosyl-L-ascorbic acid, cyclodextrin glucosyltransferase, *E. coli*, heterologous expression, l-ascorbic acid

## Abstract

In this study, the cgt gene *my*20, which encodes cyclodextrin glycosyltransferase (CGTase) and was obtained by the metagenome sequencing of marine microorganisms from the Mariana Trench, was codon optimized and connected to pET-24a for heterologous expression in *Escherichia coli* BL21(DE3). Through shaking flask fermentation, the optimized condition for recombinant CGTase expression was identified as 20°C for 18 h with 0.4 mM of isopropyl β-D-L-thiogalactopyranoside. The recombinant CGTase was purified by Ni^2+^-NTA resin, and the optimum pH and temperature were identified as pH 7 and 80°C, respectively. Activity was stable over wide temperature and pH ranges. After purification by Ni^2+^-NTA resin, the specific activity of the CGTase was 63.3 U/mg after 67.3-fold purification, with a final yield of 43.7%. In addition, the enzyme was used to transform L-ascorbic acid into 2-*O*-α-D-glucopyranosyl-L-ascorbic acid (AA-2G). The maximal AA-2G production reached 28 g/L, at 40°C, pH 4, 24 h reaction time, 50 g/L donor concentration, and 50 U/g enzyme dosage. The superior properties of recombinant CGTase strongly facilitate the industrial production of AA-2G.

## Introduction

Cyclodextrin glycosyltransferase (CGTase), also called 4-α-D-(1,4-α-D-glucanol)-transferase (EC 2.4.1.19), is a member of the α-amylase family (glycosyl hydrolase family 13) (Han et al., 2014). As a multifunctional enzyme, CGTase catalyzes four types of reactions, including hydrolysis reaction and three types of transglycosylation reactions (i.e., disproportionation, cyclization, and coupling). The cyclization reaction, which is the characteristic reaction of CGTase, is used to produce cyclodextrins. α-CD, β-CD, and γ-CD are the major structures of cyclodextrins (CD) (Leemhuis et al., 2010; Tao et al., 2020). Disproportionation and coupling reactions format glycosylated derivatives by transferring maltooligosaccharides to a variety of receptors (Fenelon et al., 2015). CGTase can produce high value-added products, such as CD and 2-*O*-α-D-glucopyranosyl-L-ascorbic acid (AA-2G). Thus, an increasing number of researchers focus on CGTase, and its demand is gradually increasing.

L-Ascorbic acid (L-AA, i.e., vitamin C) is an essential nutrient for the human body, and plays an important role in antibody and collagen syntheses, scurvy treatment, protein synthesis, and skin whitening; consequently, it has a wide application in the food, cosmetic, and pharmaceutical industries (Naidu, 2003; Han et al., 2012). However, the C2 hydroxyl group of L-AA is extremely unstable and oxidizes easily, especially in aqueous solution, thus limiting its application potential. In recent years, researchers have applied biological and chemical methods to produce a variety of L-AA derivatives that achieve better oxidation stability. Among a variety of derivatives, which include ascorbyl glucoside and ascorbyl phosphate (Mima et al., 1970; Tai et al., 2017), AA-2G has drawn enormous interest from researchers, because of its superior properties, excellent oxidation resistance, and its simple transformability to D-glucose and L-AA by α-glucosidase (Yamamoto et al., 1990). Thus, AA-2G has potential applications in the food, cosmetics, and husbandry industries. AA-2G can also enhance human immunity, as well as prevent and treat the common cold.

At present, the major method for producing AA-2G is enzymatic transglycosylation catalysis *via* several different glucosyltransferases, i.e., α-glucosidase, α-amylase, α-isomaltosyl glucosaccharide-forming enzyme, sucrose phosphatase, and CGTase (Aga et al., 1991; Bae et al., 2002; Gudiminchi and Nidetzky, 2017; Li et al., 2017). Toyoda also used the method of chemical synthesis to produce AA-2G, but the results identified the synthesis process as complicated and associated with high cost; thus, making enzymatic production unattractive (Toyoda et al., 2004). Because of the higher substrate specificity and production, CGTase is generally assumed as the most effective enzyme for AA-2G production (Jun et al., 2001). In the process of CGTase biotransformation of AA-2G by intermolecular transglycosylation, CGTase catalyzes the transfer of a glucose residue from a glucosyl donor to the C2 hydroxyl group of L-AA. Therefore, an α-configured glucoside is needed as the donor substrate in the process of enzyme reaction. Various starch-derived oligosaccharides can be used as glucosyl donor, such as cyclodextrin, starch, and maltodextrin. Currently, a number of researchers attempt to find an economic and affine glycosyl donor to synthesize AA-2G. For instance, Aga et al. (1991) used α-CD as donor and found that the conversion rate of L-AA can reach 40%; 154.8 g/L AA-2G was obtained. However, because of its high cost, α-CD is unsuitable for production of AA-2G. Therefore, Zhang et al. (2011) used β-CD as donor and achieved a AA-2G yield of 13.5 g/L. Liu et al. (2013) used maltose as donor and achieved a AA-2G yield of 1.12 g/L at optimal conditions.

Although natural CGTase is suitable for AA-2G synthesis, the conversion rates are low. To improve the conversion rate, this study applied site-directed mutagenesis to alter the glucosyl donor-binding sites of CGTases (Chen et al., 2017; Tao et al., 2019), including +1 subsite, +2 subsite, and –3 subsite. For example, Han et al. (2013) used maltose as donor and obtained higher AA-2G yield by mutant K47L, which was 64% higher than that of wild-type CGTase. In the present work, the novel recombinant CGTase MY20 was characterized and its ability to synthesize AA-2G was studied. This enzyme offers great advantages with regard to thermal stability and pH stability, which will be of great significance for the industrial production of AA-2G.

## Materials and Methods

### Materials

*my20* gene (GenBank accession no. MW561626) originated from the metagenome sequencing of marine microorganisms in the Mariana Trench. The gene *my20* was codon optimized, synthesized, and cloned into pET-24a(+) by Genewiz (Wuhan, China). *Escherichia coli* BL21(DE3) was used for heterologous expression. Luria–Bertani (LB) medium was used to cultivate *E. coli* strains. AA-2G was obtained from DB (Shanghai, China). Ni^2+^-NTA HP resin was purchased from General Electric Company (United States). Other reagents were obtained from Shanghai Sinopharm Co., Ltd. (Shanghai, China).

### Expression of the Recombinant CGTase

The recombinant plasmid was transformed into *E. coli* BL21(DE3) to express the protein. DNA sequencing (Sangon Biotech, Shanghai, China) confirmed that the target gene was inserted into the plasmid. The recombinant expression strain was inoculated into 5 ml LB medium with the appropriate concentration of kanamycin (37°C, 200 r/min, overnight). This seed solution was inoculated into 50 ml LB medium until the OD_600_ reached 0.6–0.8. Then, 0.2 mM of isopropyl β-D-L-thiogalactopyranoside (IPTG) was added to induce expression at 25°C and 200 r/min. The bacterial cells were obtained by centrifugation at 8,000 r/min at 4°C for 10 min and were resuspended in 20 mM PBS (pH 7.0). The cells were ultrasonically destroyed in an ice bath for 20 min. The cell disruption solution was centrifuged at 8,000 r/min at 4°C for 20 min, and the crude protein solution was obtained as the supernatant. In addition, to increase the expression of CGTase, with all of the above conditions unchanged, the IPTG concentration, induction time, and induction temperature were optimized to acquire high-level strains.

### Purification and SDS-PAGE

The recombinant CGTase was purified by Ni^2+^-NTA resin column. After loading into the column, the crude protein was washed with 20 mM phosphate-buffered saline (PBS; pH 7) and then, imidazole (0–500 mM) in 20 mM PBS (pH 7) was used to elute the target protein. The obtained protein was dialyzed overnight in 20 mM PBS solution to remove imidazole. Sodium dodecyl sulfatepolyacrylamide gel electrophoresis (SDS-PAGE, 12% gel) was used to analyze the purified recombinant protein.

### Cyclodextrin Glycosyltransferase Activity Assay

The methyl orange method was used to determine the CGTase activity (Sun et al., 2015). A total of 900 μl of 4% soluble starch solution dissolved in Gly-NaOH was added to the reaction vessel, and 100 μl of enzyme solution was added to react accurately at 50°C for 10 min. Then, after the reaction was terminated with 1% HCl, 4 ml of methyl orange solution (diluted fourfold) was added. The reaction solution was kept at room temperature for 20 min, and the production of cyclodextrin was estimated at an absorbance of 502 nm. One unit of activity was defined as the amount of enzyme converting 1 μmol of cyclodextrin per min.

### Biochemical Properties of the Purified Recombinant CGTase

*The optimal temperature of the recombinant enzyme was determined to be 10–100°C for 10 min. The thermal stability of recombinant CGTase was measured by placing it at different temperatures (10–90°C) for 2 h*, *and then*, *the enzyme activity was determined at 80°C. The optimum activities of recombinant CGTase were set to 100%.*

The optimal pH of recombinant activity was determined at 80°C for 10 min with the following buffers: NaAc–HAc buffer (pH 3–6), Na_2_HPO_4_–NaH_2_PO_4_ buffer (pH 6–8), Gly-NaOH buffer (pH 8–10), and Na_2_HPO_4_–NaOH (pH 10–11). To assess the pH stability, both enzyme and buffer were diluted (pH 3–11) in a proper proportion at 4°C for 12 h, and then, the enzyme activity was determined at 80°C and pH 7.

The effects of metal ions and chemical reagents on the recombinant CGTase were determined. To determine their remaining activity, the recombinant CGTase was incubated with Fe^2+^, K^+^, Ca^2+^, Mn^2+^, Ba^2+^, Cu^2+^, Mg^2+^, Li^+^, and EDTA at a final concentration of 5 mM for 2 h. The activity without metal ions and chemical reagents was used as control (100%).

### Synthesis of AA-2G

The enzymatic synthesis of AA-2G followed the method described in Tao et al. (2018). Briefly, 0.5 g L-AA and β-CD were accurately weighed and dissolved into 5 ml water. Ten percent NaOH was added to adjust the pH to 5, and then, the recombinant enzyme solution was purified (enzyme solution was added according to the content of cyclodextrin) to generate AA-2G. Then, 10 g/L Na_2_SO_3_ was added to control oxygen. Finally, distilled water was added to 10 ml. With the above conditions unchanged (40°C and pH 5), various substrate types (α-CD, β-CD, γ-CD, and soluble starch), pH levels (3, 4, 5, 6, 7, and 8), temperatures (20, 30, 40, 50, and 60°C), enzyme concentrations (25, 50, 75, 100, and 125 U/g β-CD), donor concentrations (20, 30, 40, 50, and 60 g/L), and reaction times (6, 12, 18, 24, and 30 h) were tested to identify the optimal conditions for increasing the production of AA-2G.

An Agilent 1260 High Performance Liquid Chromatography (HPLC) system with kromasil 100-5C18 column (4.6 mm × 250 mm) was used to assess the quantity of AA-2G present in the reaction solution described above. In this work, the mobile phase was 20 mM H_3_PO_4_ at an 0.8-mL/min flow rate, the column temperature was 25°C, and the PDA detector used 242 nm. The peak area of the standard sample AA-2G was used to calculate the AA-2G yield.

### Kinetic Parameters

The kinetic parameters for soluble starch were determined at 80°C and pH 7 with different concentrations of soluble starch. The Hanes-Woolf method was used to calculate *K*_*m*_ and *V*_*max*_. The kinetic parameters of AA-2G synthesis were determined at 40°C0 and pH 5. Different concentrations of β-CD (0.5, 1, 5, 10, and 25 g/L) and L-AA (1, 5, 10, 25, and 50 g/L) were used as substrates. The detailed reaction process is the same as presented in Section “Synthesis of AA-2G”. The experimental results are in accordance with the ping-pong mechanism, and the equation is as follows: *v* = *V*_max_*ab*÷(*K*_*mA*_*b* + *K*_*mB*_*a* + *ab*), where *V* represents the reaction rate (i.e., the amount of AA-2G synthesized per milligram enzyme per minute; U/mg), *V*_*max*_ represents the maximum reaction rate (U/mg), *a* represents the concentration of L-AA, *b* represents the concentration of β-CD, *A* is L-AA, and *B* is β-CD.

## Results

### Expression of Recombinant CGTase in *E. coli* BL21(DE3)

**FIGURE 1:**
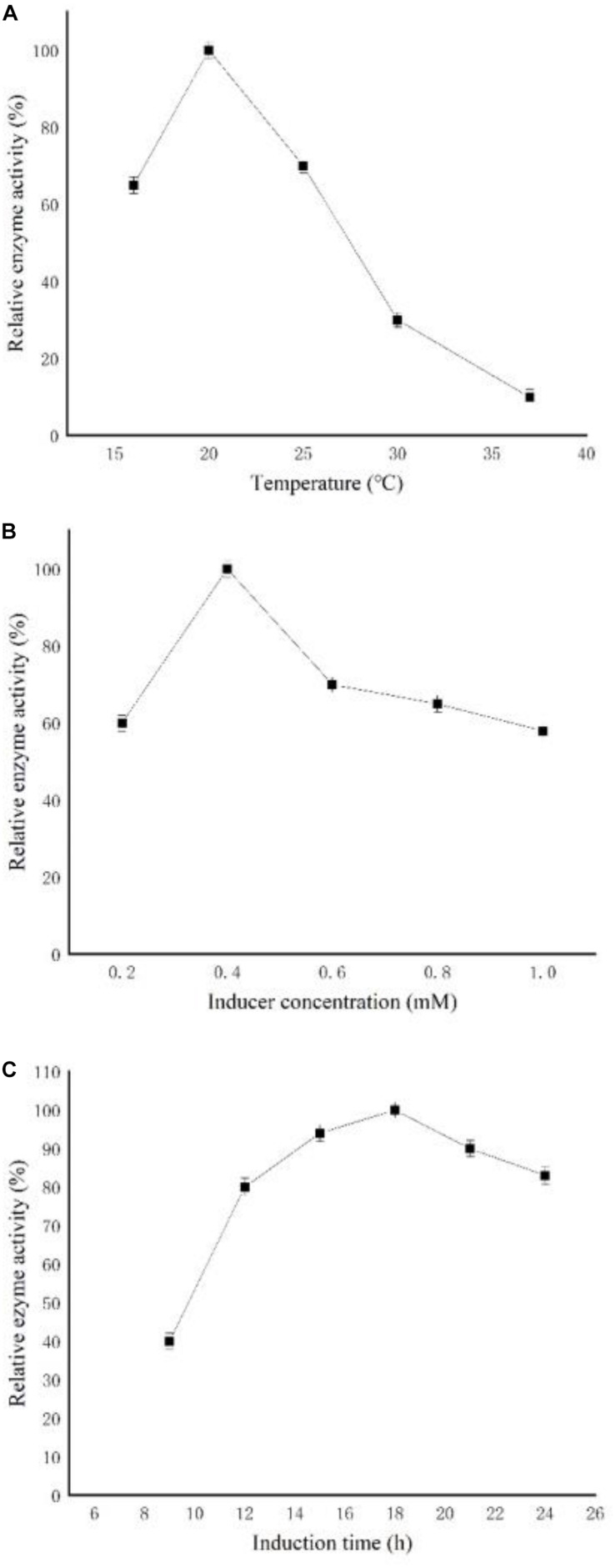
Effects of induction conditions on the expression of recombinant cyclodextrin glycosyltransferase (CGTase). **(A)** Induction temperature, **(B)** isopropyl β-D-L-thiogalactopyranoside (IPTG) concentration, and **(C)** induction time.

To increase the expression of CGTase, the culture conditions, such as induction temperature, induction time, and inducer concentration, were optimized. As shown in Figure 1A, the enzymatic activity reached the highest level at 20°C and decreased gradually with further increasing temperature. The optimal IPTG concentration was 0.4 mM, and both too low and too high IPTG concentration [which may be caused by cell death and protein degradation (Chu et al., 2018)] decreased the level of CGTase expression (Figure 1B). In addition, the key factor of induction time was tested from 6 to 24 h (Figure 1C). The result showed that the enzymatic activity reached the highest level after 18 h of induction. Based on the above results, the optimized conditions for CGTase expression were 20°C for 18 h with 0.4 mM of IPTG, after fermentation, and the OD_600_ reached 6.

### Purification and Biochemical Properties of Recombinant CGTase

**FIGURE 2:**
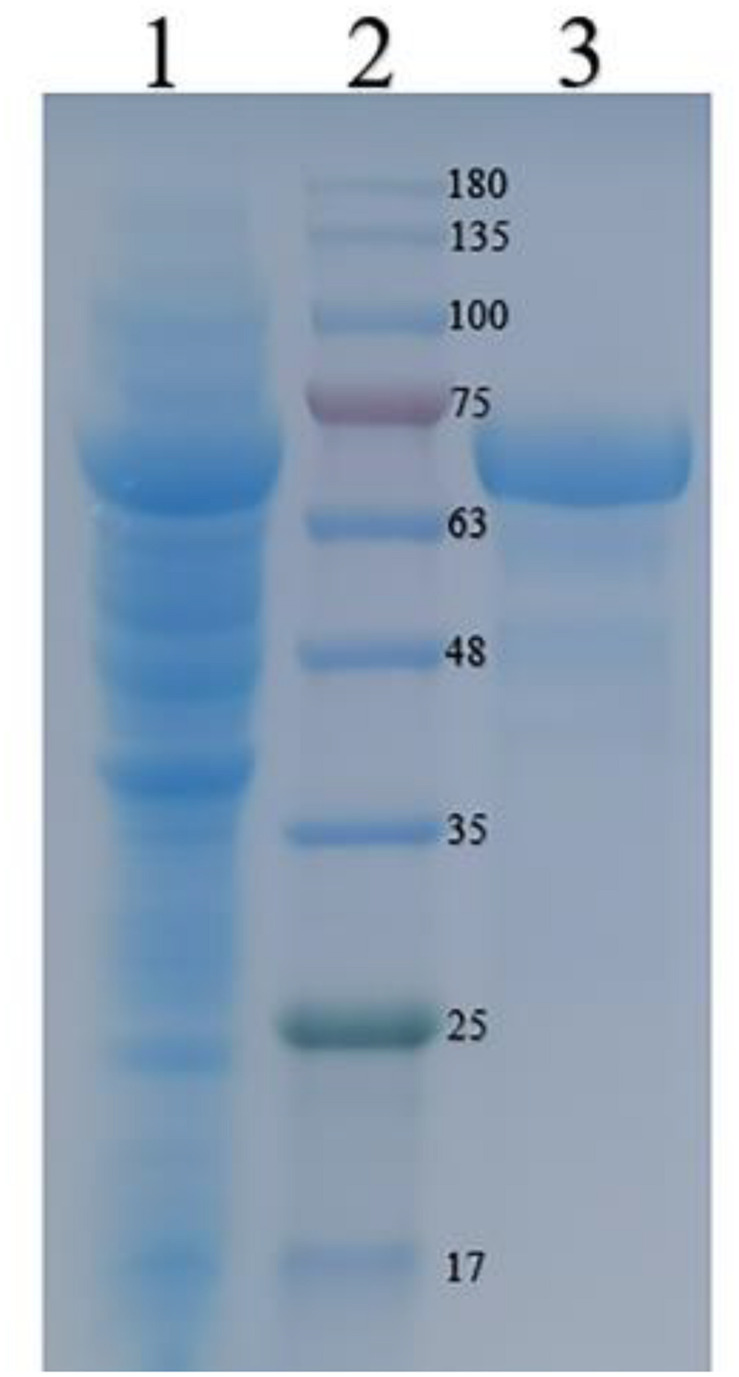
Sodium dodecyl sulfatepolyacrylamide gel electrophoresis (SDS-PAGE) analyses of the recombinant CGTase on a 12% gel. *Lane 1* crude CGTase, *lane 2* protein marker, and *lane 3* purified CGTase.

The recombinant CGTase was purified by Ni^2+^-NTA affinity chromatography. Table 1 shows the specific activity of CGTase at each purification step. After the production of Ni^2+^-NTA, the specific activity of the CGTase was 63.3 U/mg after 67.3-fold purification, achieving a final yield of 43.7%. Figure 2 shows a single band of purified recombinant enzyme on SDS-PAGE. The molecular weight of purified recombinant enzyme was about 74.3 kDa, which is consistent with the theoretical molecular mass.

**TABLE 1:** Purification of recombinant cyclodextrin glycosyltransferase (CGTase).

	Total activity (U)	Protein concentration (mg/ml)	Specificity activity (U/mg)	Purification (fold)	Yield (%)
Supernatant	160	8.5	0.94	1	100
Ni^2+^-NTA	70	0.13	63.3	67.3	43.7

**FIGURE 3:**
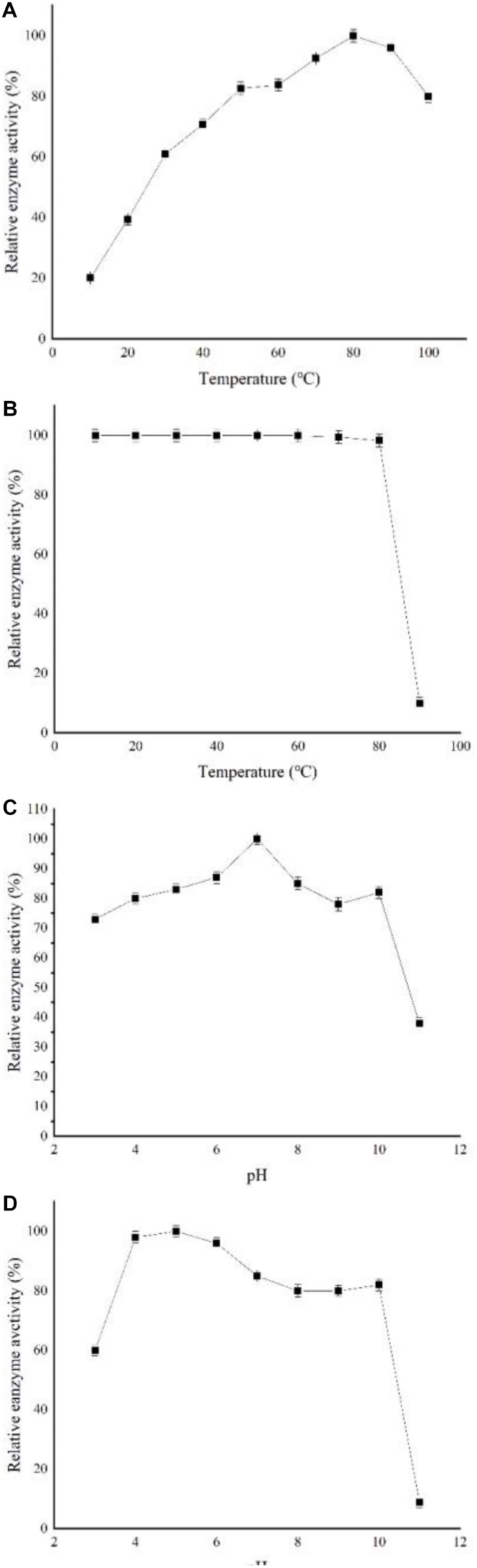
Characterization of recombinant CGTase. **(A)** Optimum temperature, **(B)** temperature stability. Reaction mixtures contained 900 μl soluble starch and 100 μl CGTase in Na_2_HPO_4_-NaH_2_PO_4_ buffer at pH 7 from 10 to 100°C for 10 min, **(C)** optimum pH, and **(D)** pH stability. Reaction mixtures contained 900 μl soluble starch and 100 μl CGTase in different buffers at pH 3–11 and were incubated at 80°C for 10 min.

Figure 3A shows that the optimal cyclization temperature of recombinant enzyme was 80°C at pH 7. At 30°C, the recombinant enzyme still exhibited more than 60% cyclization activity. The recombinant CGTase activity barely changed after treatment at a temperature range of 40–80°C for 2 h, indicating good heat resistance. However, the recombinant enzyme retained less than 10% of its activity after treatment at 90°C (Figure 3B). The optimum reaction pH of the recombinant enzyme was pH 7 at optimal temperature, and the enzyme showed high activity at a range of pH levels (Figure 3C). However, at a pH of 11, the enzyme only retained less than 50% activity. Figure 3D shows the stability of cyclization activity of recombinant enzyme in the pH range of 3–11. The recombinant enzyme could still retain more than 80% enzyme activity after preservation at pH 4–10 for 12 h, whereas its stabilities decreased clearly at pH 3 or 11, where it only retained 60 and 10% activity levels, respectively.

The effects of metal ions and chemical reagents on the activity of recombinant enzyme are shown in Table 2. K^+^, Mg^2+^, Ba^2+^, Mn^2+^, and Li^+^ exerted little effect on the recombinant enzyme, whereas Fe^2+^ and Cu^2+^ inhibited the activity. In addition, 5 mM of Ca^2+^ could improve 10% of the enzyme activity of recombinant CGTase. The chemical agents of EDTA slightly activated the cyclization activity.

**TABLE 2:** Effects of metal ions and chemical reagents on CGTase activity.

Metal ions and chemical reagents	Control	K^+^	Ca^2+^	Mn^2+^	Ba^2+^
Relative activity (%)	100	94.4 ± 1.2	110.3 ± 2.3	96 ± 3.5	96 ± 2.7
Metal ions and chemical reagents	Fe^2+^	Mg^2+^	Li^+^	Cu^2+^	EDTA
Relative activity (%)	73.9 ± 3.0	98.5 ± 1.7	98.1 ± 1.6	63.6 ± 4.3	106.7 ± 3.1

### Biosynthesis and Analysis of AA-2G

**FIGURE 4:**
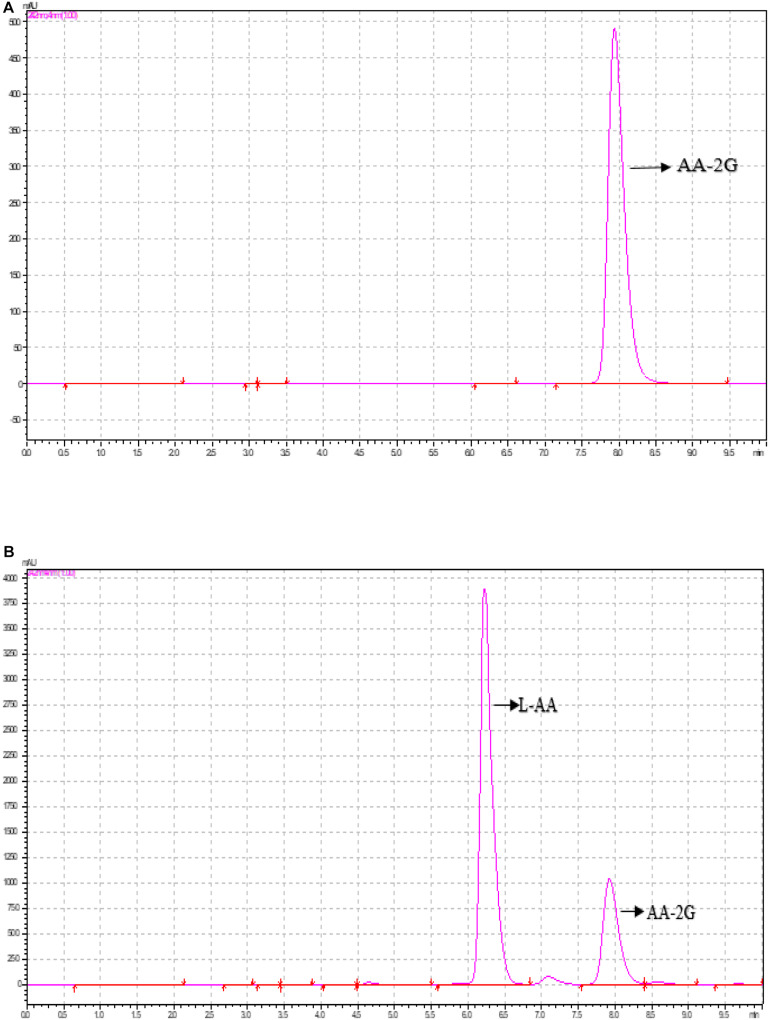
HPLC analysis of 2-*O*-α-D-glucopyranosyl-L-ascorbic acid (AA-2G) production by the recombinant CGTase. **(A)** Standard sample of AA-2G, **(B)** reaction mixture.

The standard AA-2G curve with the concentrations of 100, 200, 300, 400, and 500 mg/ml was prepared with ultrapure water. The peak time of the AA-2G standard is shown in Figure 4A. Figure 4B shows that the peak position of AA-2G was 7.9 min, which was consistent with the standard sample, and the peak position of AA-2G emerged after L-AA. Recently, CGTase has been shown to be able to use numerous oligosaccharides and polysaccharides as substrates for the transformation into AA-2G. This study used the low-cost β-CD as donor and L-AA as receptor to synthesize AA-2G. Reaction conditions, including enzyme concentration, pH, temperature, time, and donor concentration, were optimized to improve the yield of AA-2G.

**FIGURE 5:**
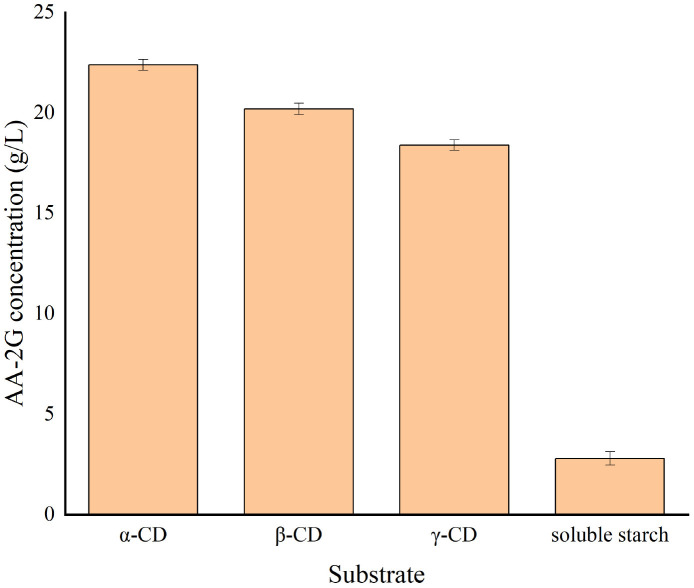
Influence of substrate species on AA-2G synthesis.

**FIGURE 6:**
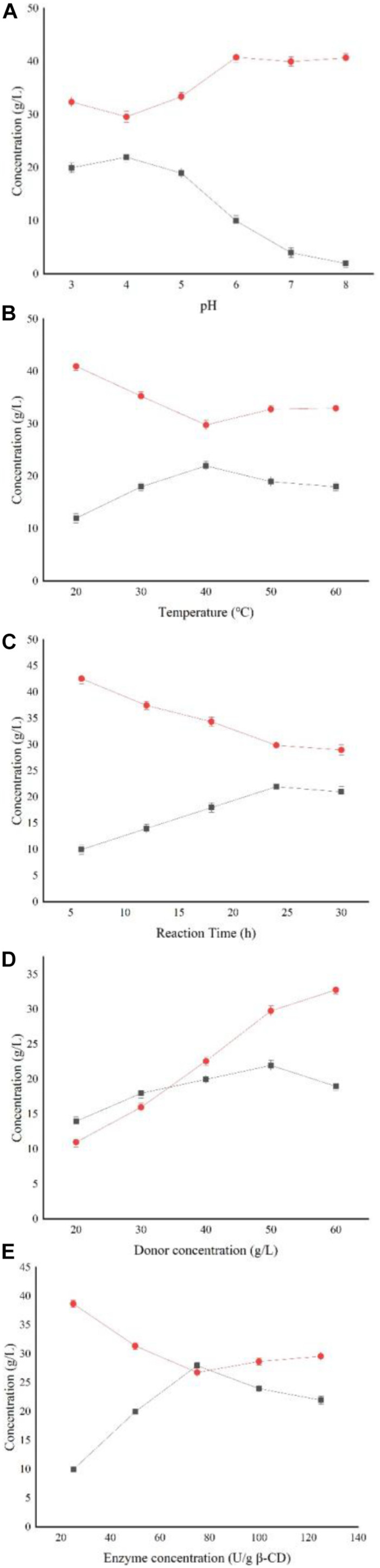
Effect of reaction conditions on AA-2G synthesis. **(A)** Influence of pH on AA-2G synthesis, **(B)** influence of temperature on AA-2G synthesis, **(C)** influence of reaction time on AA-2G synthesis, **(D)** influence of donor concentration on AA-2G synthesis, and **(E)** influence of enzyme concentration on AA-2G synthesis. AA-2G production (*filled squares*), L-AA residual content (*filled circles*).

Figure 5 shows that the yield of AA-2G is highest when α-CD is used as substrate; however, considering its industrial cost, β-CD was selected as the substrate for subsequent research. Figure 6A shows the impact of the pH level on AA-2G production. At pH 4, the yield of AA-2G was highest, and the residue of L-AA was least. The low yield of AA-2G in alkaline environment may be related to the instability of L-AA. Figure 6B shows the effect of temperature on AA-2G production. The optimum temperature for the formation of AA-2G is 40°C. With increasing temperature, the amount of AA-2G gradually decreases. The residual amount of L-AA decreased first and then increased with increasing temperature. The effects of reaction time and donor concentration on AA-2G production are shown in Figures 6C,D, respectively. With increasing reaction time, the yield of AA-2G gradually increased, and when the reaction time reached 24 h, the highest yield of AA-2G was obtained. The residual amount of L-AA decreased gradually and remained stable after 24 h. The best donor concentration of β-CD was 50 with 50 g/L VC, as shown in Figure 6D. With increasing β-CD, the yield of AA-2G also increased. This demonstrated that increased donor substrate concentration drove the overall synthesis toward the oligoglucosylation of AA-2G (Xiong et al., 2015; Gudiminchi et al., 2016). When the substrate concentrations continued to increase, the yield of AA-2G decreased. As shown in Figure 6E, the optimal enzyme dosage was 75 U/g, and with increasing amount of enzyme, the yield of AA-2G decreased gradually. When the production of AA-2G was highest, the consumption of L-AA was also highest. In conclusion, the highest yield of AA-2G was 28 g/L and was achieved at 40°C, pH 4, 75 U/g enzyme dosage, 50 g/L donor concentration, and 24 h reaction time.

### Kinetic Parameters

**FIGURE 7:**
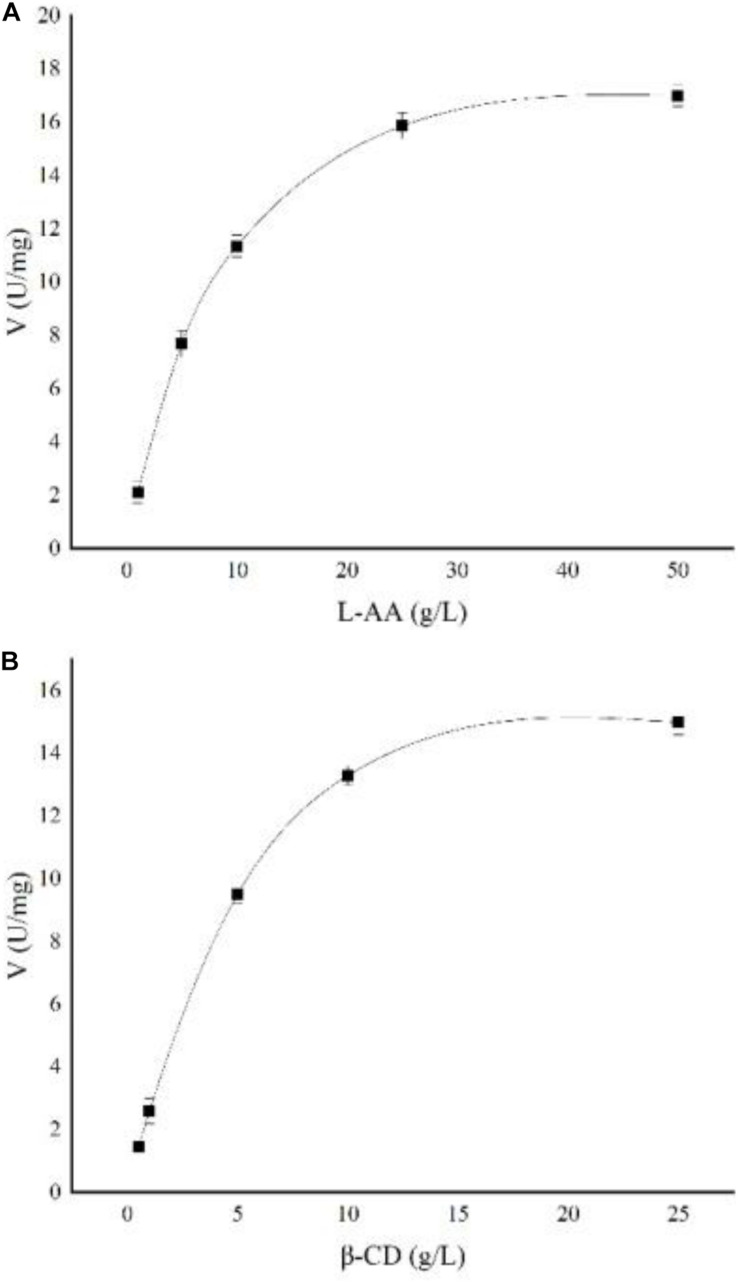
Nonlinear regression of AA-2G synthesis by recombinant CGTase. **(A)** L-AA, **(B)** β-CD.

The kinetic parameters of soluble starch were calculated as follows: *V*_*max*_ = 0.62 ± 0.04 μmol/min, *K*_*m*_ = 5.1 ± 0.09 g/L, and *K*_*cat*_ = 28.5 s^–1^. The kinetic parameters of AA-2G synthesis by recombinant CGTase were studied. As shown in Figure 7, the effects of L-AA and β-CD concentrations on the synthesis of AA-2G by recombinant CGTase were explored. The experimental data were fitted to a ping-pong mechanism, which is consistent with the experimental results of Tao et al. (2018). The kinetic parameters of the recombinant enzyme were calculated as follows: *V*_*max*_ = 21.6 ± 0.13 U/mg, *K*_*m*_*_*A*_* = 9.2 ± 0.06 g/L, *K*_*m*_*_*B*_* = 6.9 ± 0.04 g/L, and *K*_*cat*_ = 99,105 h^–1^.

## Discussion

Fermentation conditions such as induction time, inducer concentration, and induction temperature affect the expression of foreign protein (Berry, 1996; Fakruddin et al., 2013; Jaliani et al., 2014). To reduce the formation of inclusion bodies, the induction temperature mostly ranges between 15 and 25°C (Song et al., 2012), which is consistent with the results of the present study. A too low inducer concentration will lead to insufficient induction, which will reduce protein expression (Pan and Malcolm, 2000; Mora et al., 2012; Gupta and Shukla, 2016). However, excessive IPTG will lead to the formation of inclusion bodies and have toxic effects on cells (Graslund et al., 2008; Jia and Jeon, 2016); therefore, it is necessary to avoid excessive IPTG accumulation. However, the recombinant enzyme is an intracellular enzyme that needs to be broken. Its use in industrial production will increase the cost. Therefore, the yeast expression system or the *Bacillus subtilis* expression system will be considered to secrete the enzyme into the medium in future research.

The recombinant enzyme was purified by Ni^2+^ column. The specific activity of the recombinant enzyme was 66.3 U/mg, which was higher than that of a number of reported CGTases, such as *Bacillus* sp. 8SB, *Bacillus* sp., *alkalophilic Bacillus* sp. g-825-6. The specific activities were 48, 2.24, and 5.57 U/mg, respectively (Hirano et al., 2006; Kitayska et al., 2011; Mora et al., 2012). The molecular weight of the recombinant enzyme is similar to that of other CGTases, such as *Bacillus* sp. Y112 (74 kD), *Bacillus megaterium* (66 kD), and *Bacillus pseudoalcaliphilus* (75 kD) (Vazquez et al., 2016; Zhang et al., 2017; Li et al., 2020).

The recombinant enzyme achieved good physical and chemical properties, which proved that the enzyme has promising industrial application value. The properties of CGTase from different sources are listed in Table 3. With regard to thermal stability, the optimal temperatures of a number of reported CGTases range between 40 and 60°C. The optimal temperature of the recombinant CGTase encoded by the *my*20 gene is 80°C. The most important property of the enzyme is its thermal stability. The activity of most CGTases will decrease or even be completely inactivated when the temperature exceeds 50°C for a period of time. The stability of the recombinant CGTase encoded by the *my20* gene is much better than these recombinant CGTases. Because of the good thermal stability of the recombinant enzyme, the application of the recombinant enzyme for industrial production will extend its working time, reduce the pollution risk, and improve the solubility of the substrate (Zhang et al., 2018). Therefore, the recombinant enzyme will have great potential in the industrial production of CD and AA-2G. As shown in Table 3, the optimal pH of CGTase reported in this study ranged from 6 to 8. However, a number of CGTase exist whose optimum pH is alkaline, such as *Bacillus* sp. Y112 with an optimum pH of 10 (Li et al., 2020). In the present study, the optimum pH was 7, and the CGTase encoded by MY20 achieved better pH stability than the enzyme (Table 3). When CGTase (Table 3) is exposed to pH 4–5 for a certain period of time, its activity will be greatly decreased. When CGTase was used for the production of AA-2G, the yield of AA-2G may be reduced, because most of the experimental data show that the recombinant CGTase can produce more AA-2G in an acidic environment. However, the recombinant CGTase encoded by MY20 showed good acid tolerance. Therefore, because of its good thermal and pH stabilities, the recombinant enzyme will have great significance for guiding the industrial production of AA-2G. With regard to the tolerance to metal ions, Ca^2+^ could increase the activity of recombinant enzyme, which is consistent with the results of *Paenibacillus illinois* ZY-08 (Lee et al., 2013). The results of *Paenibacillus pabuli* US132 also showed that Fe^2+^ and Cu^2+^ could reduce the enzyme activity (Jemli et al., 2007).

**TABLE 3:** Properties of CGTase from different strains.

Strain	Source	Optimal temperature	Optimal pH	*K*_*m*_ (g/L)	References
*Paenibacillus illinoisensis* ZY-08	Soil in China	40°C	pH 7	0.48	Lee et al., 2013
*Paenibacillus pabuli* US132	Tunisian soil	60°C	pH 6.5	8.62	Jemli et al., 2007
*Alkaliphilic Bacillus* sp. SD5	Lake Salda in Burdur Province	50°C	pH 6	13.59	Kabacaoglu and Karakas, 2017
*Bacillus pseudalcaliphilus* 8SB	Soil from Sapareva Bania region	60°C	pH 6	Not mentioned	Kitayska et al., 2011
*Bacillus lehensis* CGII	Wastewater samples from a cassava flour mill in Brazil.	55°C	pH 8	Not mentioned	Blanco et al., 2014
*my*20	Mariana trench	80°C	pH 7	5.1	This study

As generally accepted, a smaller *K*_*m*_ implies a higher affinity of enzyme to substrate. In this study, where soluble starch was used as substrate, the *K*_*m*_ value of recombinant enzyme was 5.1 g/L, which was lower than that of CGTase from *Bacillus lehensis* CGII and *alkaliphilic Bacillus* sp. SD5, as shown in Table 3.

2-*O*-α-D-glucopyranosyl-L-ascorbic acid, as the best substitute of L-AA, has received wide interest of researchers. In recent years, reports on the synthesis of AA-2G by CGTase are becoming more numerous, as shown in Table 4. Many studies have shown that cyclodextrins (α-CD, β-CD, and γ-CD), soluble starch, and maltose can be used as donor substrates for the synthesis of AA-2G. Although the achieved yield of AA-2G is very high, using α-CD as donor is expensive and therefore, not suitable for industrial production. When using γ-CD and soluble starch as substrate, the yield of AA-2G is low and the price of γ-CD is also high. The experimental results of the present study are consistent with those of Jiang et al. (2018). They used soluble starch as substrate and the achieved AA-2G yield was 61% lower than that of α-CD, and 36% lower than that of β–CD (Jiang et al., 2018). Therefore, in this study, β-CD was selected as the donor substrate to synthesize AA-2G. The optimum pH for AA-2G production is pH 4–6, which is consistent with the results of the present study. However, there is also a CGTase from *Bacillus* sk 13.002, with an optimal pH for AA-2G synthesis of ∼8 (Eibaid et al., 2014). However, the yield of AA-2G synthesis of the recombinant CGTase encoded by MY20 decreased significantly at pH 8. This may be caused by the instability of L-AA aqueous solution in the alkaline environment. The optimum temperature for synthesis of AA-2G is mostly between 36 and 50°C, which is consistent with the results of the present study. When the reaction temperature increased, the yield of AA-2G decreased. This may be due to the unstable oxidative decomposition of L-AA at high temperature (Teng et al., 2018), or the decrease of coupling activity and disproportionation activity of recombinant enzyme at high temperature. In this study, the optimal reaction time of AA-2G synthesis is 24 h, which is consistent with the results of Gudiminchi et al. (2016). They used α-CD as substrate, and the yield of AA-2G did not increase after 24 h (Gudiminchi et al., 2016). In summary, under optimal reaction conditions, the recombinant CGTase encoded by MY20 can produce 28 g/L AA-2G, which is higher than the amount of AA-2G synthesized by many other CGTases, as shown in Table 4. Moreover, because of the good thermal and pH stabilities, the recombinant enzyme can transform L-AA to produce more AA-2G; therefore, the recombinant enzyme will have great economic value in the industrial production of AA-2G. Current experiments show that the site-directed mutagenesis of CGTase +1 and CGTase +2 can improve the yield of AA-2G. Therefore, subsequent experiments will assess the use of site directed mutagenesis to further improve the yield of AA-2G.

**TABLE 4:** Optimum reaction conditions of 2-*O*-α-D-glucopyranosyl-L-ascorbic acid (AA-2G) synthesis by CGTase from different strains.

Strain	Temperature (°C)	pH	Donor substrate	AA-2G concentration (g/L)	References
*Paenibacillus macerans*	37	5	β-CD	15	Zhang et al.
*Paenibacillus macerans*	36	6.5	Maltose	1.12	Liu et al.
*Thermoanaerobacter* sp.	50	4.5	α-CD	143	Gudiminchi et al., 2016
*Paenibacillus* sp.	37	6.5	γ-CD	1.7	Jun et al.
*P. macerans* JFB05-01	40	5	α-CD	9.1	Jiang
*my20*	40	4	β-CD	28	This study

This study showed that the recombinant enzyme encoded by *my*20 gene offers great advantages in pH and temperature stabilities. On the one hand, high optimum temperature and good thermal stability are helpful to prolong the working time and increase the solubility of the substrate. On the other hand, the recombinant enzyme has strong acid resistance, which is helpful for the production of AA-2G. Therefore, the recombinant enzyme will have great application prospects for the industrial production of AA-2G.

## Data Availability Statement

The raw data supporting the conclusions of this article will be made available by the authors, without undue reservation.

## Author Contributions

KS: conceptualization, methodology, writing-original draft, and writing–review and editing. JS: formal analysis and writing-original draft. WW: data curation and methodology. JH: funding acquisition and writing–review and editing. All authors have read and agreed to the published version of the manuscript.

## Conflict of Interest

The authors declare that the research was conducted in the absence of any commercial or financial relationships that could be construed as a potential conflict of interest.
