# Supplementary material for: Heterologous Expression of Cyclodextrin Glycosyltransferase my20 in Escherichia coli and Its Application in 2-O-α-D-Glucopyranosyl-L-Ascorbic Acid Production
Source: Front Microbiol. 2021 May 28;12:664339. doi: 10.3389/fmicb.2021.664339 (PMC8195388; doi:10.3389/fmicb.2021.664339)
Supplement: Supplementary file 1 [file Data_Sheet_1.docx]

A


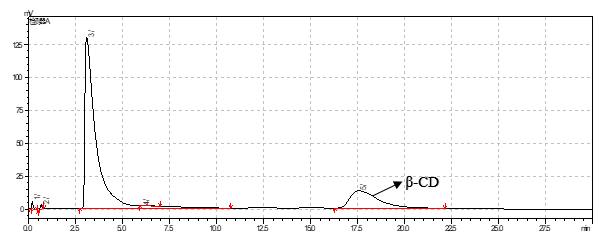


B


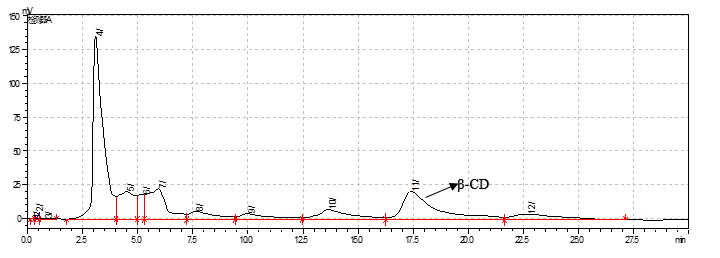


C


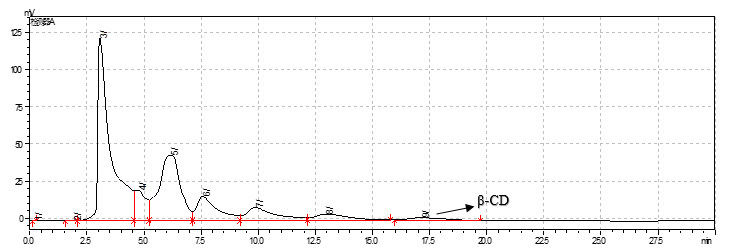


Fjgure 1: Detection of β – CD. (A): Analysis of β-CD standard by HPLC; (B): HPLC analysis of β-CD for 2 h reaction; (C): HPLC analysis of β-CD for 24 h reaction.
